# Novel Lithium-Ion Capacitor Based on a NiO-rGO Composite

**DOI:** 10.3390/ma14133586

**Published:** 2021-06-27

**Authors:** Qi An, Xingru Zhao, Shuangfu Suo, Yuzhu Bai

**Affiliations:** 1 Department of Mechanical Engineering, Tsinghua University, Beijing 100084, China; sfsuo@tsinghua.edu.cn (S.S.); baiyuzhu403@163.com (Y.B.); 2 Beijing Institute of Nanoenergy and Nanosystems, Chinese Academy of Sciences, Beijing 101400, China; zhaoxingru@binn.cas.cn

**Keywords:** lithium-ion capacitors, NiO-rGO composite, energy density, power density

## Abstract

Lithium-ion capacitors (LICs) have been widely explored for energy storage. Nevertheless, achieving good energy density, satisfactory power density, and stable cycle life is still challenging. For this study, we fabricated a novel LIC with a NiO-rGO composite as a negative material and commercial activated carbon (AC) as a positive material for energy storage. The NiO-rGO//AC system utilizes NiO nanoparticles uniformly distributed in rGO to achieve a high specific capacity (with a current density of 0.5 A g^−1^ and a charge capacity of 945.8 mA h g^−1^) and uses AC to provide a large specific surface area and adjustable pore structure, thereby achieving excellent electrochemical performance. In detail, the NiO-rGO//AC system (with a mass ratio of 1:3) can achieve a high energy density (98.15 W h kg^−1^), a high power density (10.94 kW kg^−1^), and a long cycle life (with 72.1% capacity retention after 10,000 cycles). This study outlines a new option for the manufacture of LIC devices that feature both high energy and high power densities.

## 1. Introduction

With the ongoing energy crisis and international impetus to mitigate environmental pollution and climate change, energy storage devices that serve as intermediaries of clean and efficient energy are attracting ever more attention among researchers [1,2,3,4,5,6,7]. Energy storage devices such as lithium-ion batteries (LIBs) and supercapacitors (SCs) are widely used due to their satisfactory electrochemical performance [7,8,9]. The competitive advantage of LIBs is their high energy density (i.e., 150–200 W h kg^−1^). However, their power density is fairly low (less than 100 W h kg^−1^) and a poor cycle life limits their practical applications. Meanwhile, SCs can offer high power density (10 kW kg^−1^) and excellent cycle stability, but feature low energy density (5–10 W h kg kg^−1^), impeding their utility as singular energy storage devices [10,11,12]. These differences derive from the different energy storage mechanisms of these systems; LIBs store energy by inserting lithium ions into, or extracting them from, most electrodes, whereas SCs store energy through the adsorption/desorption of ions on the electrode surface [13].

To maximize their electrochemical performance, lithium-ion capacitors (LICs) have been widely studied. The main factor affecting the performance of LICs is the mismatch of reaction kinetics between the two electrodes; the parameter with the greatest influence in this regard is the electrode material [14,15,16,17,18]. Therefore, the identification of electrode materials with a suitable specific capacity, remarkable rate performance, and excellent stability has become a key challenge. With respect to the positive electrode, carbon-derived materials (activated carbon (AC) [18], graphene [19], and metal–organic framework (MOF)-derived porous carbon [20,21], among others) are considered the best option. Notably, the most widely used is AC, which is characterized by a high specific surface area. With respect to the anode materials, the AC positive materials match the abundant micropores, and high-conductivity materials appear to be a rational choice. There are three main types of anode material: embedded materials (such as graphite and graphene [21,22,23,24,25,26]), conversion materials (e.g., Fe_3_O_4_ [27]), and alloys (including Si/Cu and SiO_2_/C [28,29]). Among these, metal oxides with a high specific capacitance have been widely used, whereas low conductivity and irreversible structural changes confine their rate index and stability [30].

Among transition metal oxides, NiO is considered to be ideal due to its excellent chemical and physical properties, such as its high theoretical capacitance and ease of processing. With respect to the quality of electrochemical properties [31,32], the synergistic relationship between graphene nanosheets and ultrafine NiO nanoparticles enables the development of materials with enhanced stability and rate performance. Regarding the electrochemical properties of NiO-rGO composite, this study found that after 200 cycles at a current density of 0.5 A g^−1^, the capacity retention rate was 95.6% and the coulomb efficiency was ~100%, indicating good cycle stability. The large specific surface area and adjustable pore structure of AC have made it the pre-eminent choice of positive material. Therefore, AC has been introduced alongside NiO-rGO for the construction of positive and negative materials for LICs. In this study, an LIC was rationally designed using a NiO-rGO composite as the negative material and commercial AC as the positive material. Additionally, a NiO-rGO//AC LIC system with a mass ratio of 1:2 to 1:4 (negative/positive) was successfully fabricated. LICs with a mass ratio of 1:3 achieved a 122 W h kg^−1^ energy density, a 32.3 kW kg^−1^ power density, and a capacity retention of 72.1% after 10,000 cycles.

## 2. Experiment

### 2.1. Synthesis of GO

The graphite oxide utilized in this experiment was prepared by means of the traditional Hummer method. First, graphite powder (2 g), NaNO_3_ (2 g), and concentrated H_2_SO_4_ (92 mL) were mixed and stirred for 4 h. Next, 12 g of KMnO_4_ was added and stirred at 25 °C for 2 h. Then, 184 mL of deionized water was then slowly added to induce a full reaction. After that, 40 mL of H_2_O_2_ was added, and stirred for 15 min. Finally, it was washed, centrifuged, and dried, after which the preparation of the GO was complete.

### 2.2. Synthesis of the NiO-rGO Composite

The NiO-rGO composite was obtained by means of a simple hydrothermal reaction. First, 62 g of NiSO_4_ was dissolved in 50 mL deionized water, stirring for 15 min to fully dissolve it. Next, 4 mL of ammonia water (25%) was slowly added to the NiSO_4_ and then dispersed by ultrasound for 30 min. Through the above steps, solution A was obtained. Then, 20 mg of GO was dissolved in 50 mL deionized water. By dispersing the mixture using ultrasound for 30 min, solution B was obtained. Solution A was then slowly added to solution B and stirred for 20 min to obtain solution C. Subsequently, 8 mg mL^−1^ NaBH_4_ was carefully added to solution C, which was then stirred for 1 h and heated in a water bath at 80 °C for 12 h. The mixed solution was cooled to 25 °C, washed, and dried with deionized water. The final product was the NiO-rGO composite.

### 2.3. Characterization

The X-ray diffraction (XRD) properties of the crystal were measured using Cu Kα (λ = 0.15406 nm) radiation on a D8 focusing diffractometer (Bruker D8 DISCOVER, Bruker, Leipzig, Germany). The morphology of the materials was tested by means of a scanning electron microscope (SEM, Nova NanoSEM 450, FEI, Hillsborough, Oregon, OR, USA), energy-dispersive X-ray spectroscopy (EDX, SciAps, Boston, MA, USA), and transmission electron microscopy (TEM, JEOL JSM-2010, JEOL, Tokyo, Japan). The Raman spectra were determined using a LabRAM HR Raman spectrometer (Horiba, Kyoto, Japan). The X-ray photoelectron spectroscopy (XPS) spectra of the materials were measured using ESCALAB 250Xi (Thermo Fisher, Waltham, MA, USA).

### 2.4. Electrochemical Measurements

The coating method was used for the preparation of the electrode. The active materials, Super C45 and polyvinylidene fluoride (PVDF), were blended in an *N*-methyl-2-pyrrolidone (NMP) solution at a ratio of 8:1:1. The weight ratio of NMP was approximately 75–80%. After that, the mixed solution of negative and positive materials was evenly coated onto a collector of copper foil (negative) or aluminum foil (positive) by means of a coater. The coated foil was then cut into sheets of 10 mm (negative electrode) or 13 mm (positive electrode) after drying. A working electrode was obtained by vacuum drying the resulting product overnight at 120 °C. A button cell was assembled using lithium foil as the counter electrode in a 3-electrode system and polyethylene as a separator. The electrolyte was 1 M LiPF_6_, which was blended in a mixed solvent of dimethyl carbonate (DMC), diethyl carbonate (DEC), and ethylene carbonate at a volume ratio of 1:1:1. The mass load for a single electrode was 1 mg cm^−2^.

The LICs were assembled utilizing a pre-embedded lithium negative and pristine positive 2-electrode cell. The ratios of the mass of the active materials of the negative (NiO-rGO) and positive (AC) electrodes were 1:2, 1:3, and 1:4. Their electrochemical performance, including pre-lithiation, galvanostatic charge–discharge progress, and rate and cycle life, were measured using a Neware battery-testing device. Cyclic voltammetry (CV) curves were determined using a BioLogic VMP3 electrochemical station (Biologic, Sohn, France). The calculation process of energy density, specific capacitance, and power density (based on the total mass of the two electrode materials) was as follows:

(1) $$C=\frac{\int^{\;}IdV}{2m\Delta Vv}$$

(2) $$E=\int_{t1}^{t2}IVdt=\frac{1}{2}C(V_{\max}+V_{\min})\;(V_{\max}-V_{\min})$$

(3) $$P=\frac{E}{t}$$

where *I* is the current, *v* is the scanning speed, V is the voltage, *v* is the scanning speed used in the CV test, *t* is the discharge time, *m* is the total mass of electrode materials, and V_max_ and V_min_ are the voltage at the start and the end of discharge, respectively.

## 3. Results and Discussion

### 3.1. Material Performance Characterization

**Figure 1:**
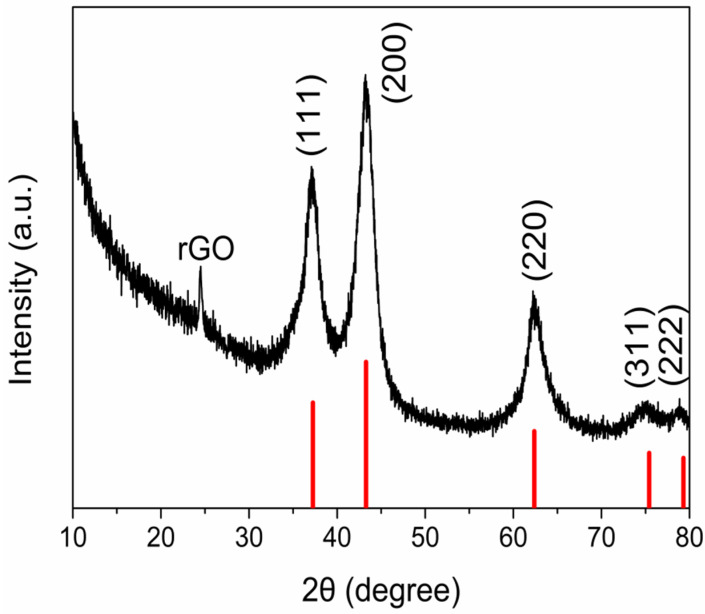
XRD pattern of the NiO-rGO composite.

In the XRD pattern (Figure 1), the diffraction peak at 24.5° was the characteristic peak of rGO. The main diffraction peaks of the NiO-rGO composite appeared at 37.2°, 43.3°, 62.8°, 75.4°, and 79.3°, corresponding to crystal plane diffractions of (111), (200), (220), (311), and (222) a.u., respectively, and aligning with NiO’s standard card, with no other peaks emerging. This further demonstrated that the NiO-rGO composite, having been prepared following the aforementioned method, had a high crystal purity, with no other substances appearing.

**Figure 2:**
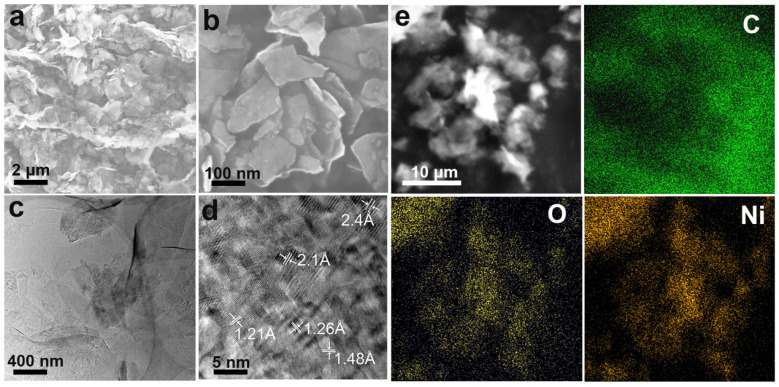
(**a**,**b**) SEM of the NiO-rGO composite. (**c**,**d**) TEM of the NiO-rGO composite. (**e**) EDX mapping (C, O, Ni) of the NiO-rGO composite.

The layered rGO was well preserved and the thickness of the layered composite was less than 10 nm (Figure 2a,b), which indicated that the number of rGO layers was small and that the rGO sheet did not agglomerate because NiO nanoparticles were uniformly anchored in it. Furthermore, there was no serious NiO aggregation and all NiO nanoparticles were well anchored to the rGO, indicating that the morphology of the composite was good. Figure 2c displays uniform NiO nanoparticles in the size of ten to tens of nanometers. Figure 2d shows that the NiO crystal size was about 5.5 ± 1.5 nm and the crystal plane spacing of NiO nanoparticles was about 0.24 nm, corresponding to the (111) plane of the cubic NiO crystal. An EDX analysis (Figure 2e) revealed that the distribution of Ni and O was relatively uniform, and that there was no obvious aggregation. Moreover, the C, Ni, and O content was 78%, 12%, and 10%, respectively. Therefore, the NiO-rGO composite could be synthesized without additional material doping.

**Figure 3:**
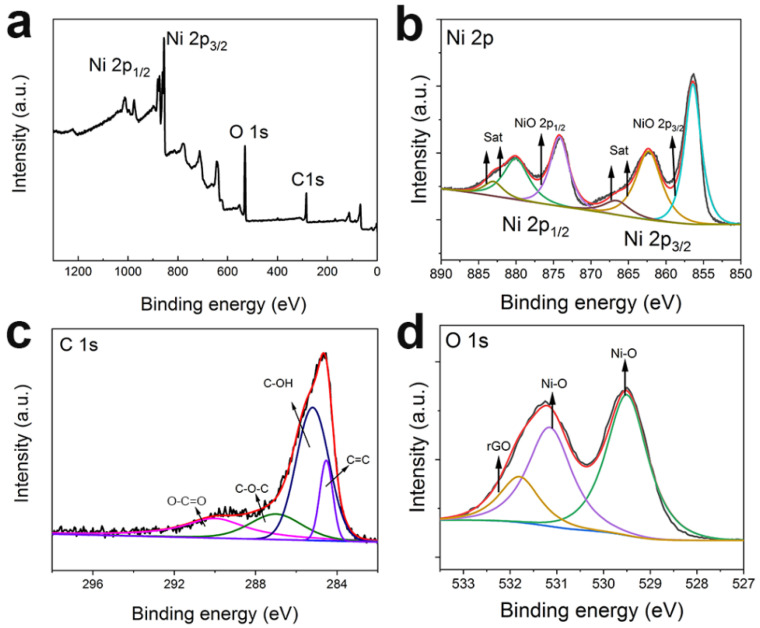
(**a**) XPS and high-resolution spectra of (**b**–**d**) Ni 2p, C 1s, and O 1s peaks in the NiO-rGO composite.

An XPS scan was used to evaluate the surface electronic states of the elements. Figure 3a displays the Ni 2p, C 1s, and O 1s peaks that confirm the coexistence of NiO and rGO in the composite. In the Ni 2p spectra (Figure 3b), the peaks at 855.8 eV and 873.6 eV belong to the Ni 2p_2/3_ and Ni 2p_1/2_, respectively. The C 1s spectra (Figure 3c) were fitted to four peaks of 284.7 eV, 285.4 eV, 287.0 eV, and 289.9 eV, corresponding to C=C, C–OH, C–O–C, and O–C=O, respectively. The O 1s spectrum (Figure 3d) showed three main peaks, with the one at 531.8 eV corresponding to rGO, and those at 531.2 eV and 529.3 eV both corresponding to NiO. Based on the above discussion, the bond between nickel and an oxygen-containing functional group may be the result of the combination of NiO nanocrystals and rGO nanoplates [33].

**Figure 4:**
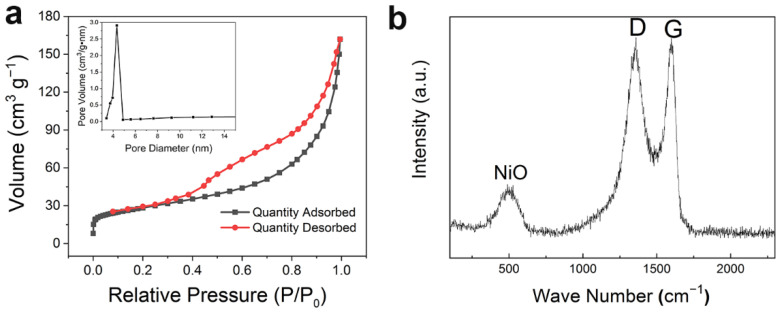
(**a**) N_2_ adsorption–desorption isotherm and pore size distribution (BJH) model of the NiO-rGO composite; (**b**) Raman spectra of the NiO-rGO composite.

Figure 4a presents the N_2_ adsorption/desorption isotherm and pore size distribution of NiO-rGO. The specific surface area of the NiO-rGO composite was 89.7 m^2^ g^−1^, lower than that of rGO (about 204.3 m^2^ g^−1^), due to the much heavier weight of the NiO compared to the rGO. The mesopores (concentrated at 4 nm) can be observed in the graph, and can be attributed to the interaction between the NiO and rGO.

The Raman spectra of the composite (Figure 4b) indicate that there were two peaks of C at 1357 cm^−1^ (D peak) and 1598 cm^−1^ (G peak), and the characteristic peak of NiO appeared at 500 cm^−1^. Researchers often use the ratio of the D to G peak (*I*_D_/*I*_G_) as the index for detecting the defect density. The *I*_D_/*I*_G_ of the NiO-rGO composite was 0.96, which indicated that abundant defects were introduced during its synthesis. The existence of defects can improve the ion/electron transport speed, improve the conductivity, and further improve the electrochemical performance.

### 3.2. Performance of Half-Cells

To evaluate the properties of the NiO-rGO composite, constant current charge and discharge were tested under 0.05–3 V (vs. Li/Li^+^).

**Figure 5:**
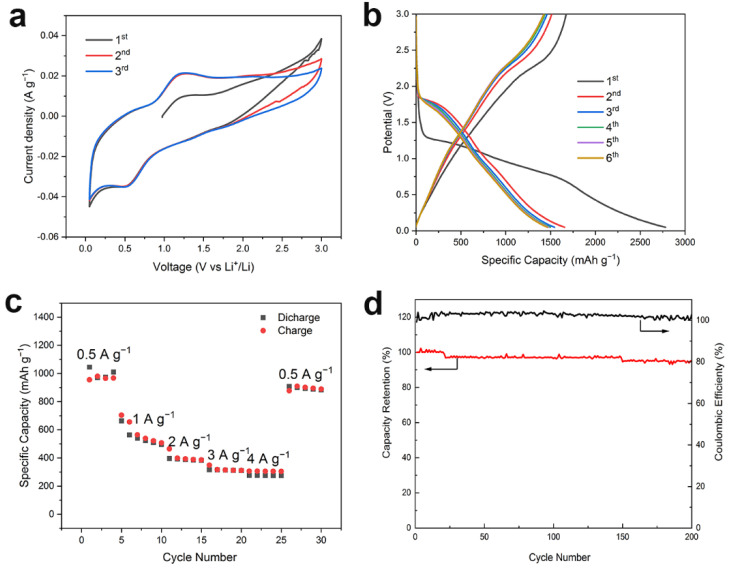
Electrochemical performance of the NiO-rGO. (**a**) CV curves at 0.1 mV s^−1^. (**b**) First four charge–discharge curves at 0.1 A g^−1^. (**c**) Rate performance. (**d**) Cyclic behavior at 0.5 A g^−1^.

Figure 5a depicts the CV curves (0.05–3 V, 0.1 mV s^−1^). During the positive process, an obvious peak appeared at ~0.5 V due to the formation of a solid electrochemical interface (SEI) film. The second and third curves essentially coincided, indicating the high stability of the material. Figure 5b shows the charge/discharge curves of the NiO–rGO composite during the first four cycles at 0.1 A g^−1^ and 0.05–3.0 V (vs. Li/Li^+^). The initial discharge capacity and charging capacity of the NiO-rGO composite were 2700 mA h g^−1^ and 1644 mA h g^−1^, respectively: higher than the theoretical capacity of NiO (718 mA h g^−1^). The formation of an SEI film during the first charge and discharge consumes a lot of lithium ions, which leads to a large difference in specific capacity between charge and discharge. In later charge and discharge processes, it gradually stabilizes. Figure 5c displays the rate performance at 0.5–4 A g^−1^. When at 0.5 A g^−1^, the charge capacity was 945.8 mA h g^−1^. This slower reaction rate is more conducive to the diffusion of lithium ions into NiO-rGO, leading to a high specific capacity. At 4 A g^−1^, the charge capacity was 275.6 mA h g^−1^, which related to the crystallinity and size of the nanocrystals. When charging and discharging at higher currents, the speed of lithium-ion intercalation and exfoliation in the electrode material is faster, and crystal lattice distortion is more likely to occur, leading to crystal shedding and electrochemical performance degradation. The charge–discharge reaction is gradually balanced when the current reverts to 0.5 A g^−1^. A capacity of 897.3 mA h g^−1^ can be obtained, which is better than, or comparable to the reported data (high specific capacity of 520 mA h g^−1^ at 2400 mA g^−1^) [34]. This shows that the NiO-rGO composite has good reversible cycle performance and high reversible capacity at high current density. Figure 5d shows that, after 200 cycles at a current density of 0.5 A g^−1^, the capacity retention rate was 95.6% and the coulomb efficiency was nearly 100%, indicating good cycle stability. Electrochemical impedance spectroscopy (EIS) was performed in the frequency range of 100 kHz to 0.01 Hz with an amplitude of 5 mV. The EIS spectrum appears as a semicircle at high frequency and has a linear part at low frequency. In the high frequency range, the intercept on the real axis is the volume solution resistance (Re), and the diameter of the semicircle on the real axis is approximately equal to the charge transfer resistance (Rct). The oblique line in the low-frequency region indicates the ion diffusion process in the electrode. As shown in Figure S1, the Re of NiO-rGO is significantly lower than that of NiO, its corresponding straight line is steeper than the one for NiO, and the ion diffusion on the surface is faster. The abovementioned excellent properties were mainly due to the presence of rGO, which not only enhances ion and electron transmission, but also reduces the structural collapse of metal oxides during the intercalation and desorption of lithium ions.

### 3.3. Electrochemical Performance of LICs

In this study, LICs were prepared using AC as the positive electrode and pre-lithiated NiO-rGO as the negative electrode, with a mass ratio of 1:2 to 1:4 (negative-to-positive). Based on this, the electrochemical performance of the LICs was optimized.

**Figure 6:**
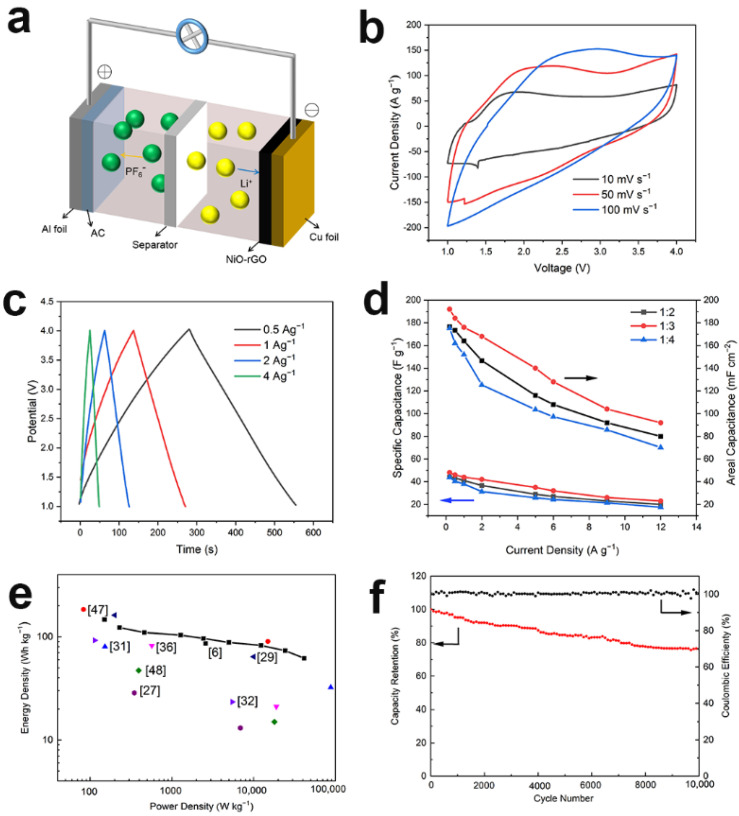
(**a**) Schematic diagram of working mechanism for the NiO-rGO//AC system. (**b**) CV curves. (**c**) Galvanostatic charge−discharge curves with a mass ratio of 1:3. (**d**) Specific capacitance (left axis) and area capacitance (right axis) of NiO-rGO//AC. (**e**) Ragone plots (mass ratio: 1:3), in comparison with recent literature. (**f**) Cycle life and coulombic efficiency curves of LICs with a mass ratio of 1:4, the inset of which was a demonstration of NiO-rGO//AC.

The charge storage mechanism is shown in Figure 6a. During charging, PF_6_^−^ ions were transported to the AC positive electrode, whereas Li^+^ ions were inserted on the surface of the NiO-rGO negative electrode. The discharging procedure was in contrast to the charging process. Figure 6b shows the CV curve of the LICs with a mass ratio of 1:3. At the scanning rates of 10, 50, and 100 mV/s^−1^, the curves were nearly rectangular, indicating that the system had attained a good capacitance performance and rate capability. The energy storage mechanism of NiO is such that with the increase in scanning speed, the rectangular shape of CV curve is difficult to maintain. Figure 6c displays the constant current charge–discharge curve under different current densities (the mass ratio was 1:3, 1.0–4.0 V). At 0.5 A g^−1^, the middle section of the charge curve was curved, which was due to the insertion and extraction of the lithium ions; at 1.5 A g^−1^, the curve exhibited triangular symmetry, which conforms to the capacitance characteristics. Furthermore, no significant IR drop occurred in these curves, indicating that the internal resistance of the system was very low. Due to the difference in the specific capacitance of positive and negative materials, in order to optimize the voltage window and obtain the best performance, it is necessary to adjust the active material weight ratio of the positive and negative electrodes. Figure 6d demonstrates the rate capability of the NiO-rGO//AC system. It can be noted that LICs with negative-to-positive mass ratios of 1:3 feature optimized performance. Moreover, the capacitance of the NiO-rGO//AC system was 43.75 Fg^−1^ (at 0.2 A g^−1^) (based on the proportion of active substances). The NiO-rGO//AC hybrid system was compared to other capacitors (Table S1). Compared to other hybrid systems, NiO-rGO//AC has major advantages, and it can achieve greater energy combination and maintain satisfactory energy density at a high power output (Figure 6e). To assess its practical applications, a cycle-life test was performed. Figure 6f indicates that the capacity retention rate of NiO-rGO//AC was 76.1% after 10,000 cycles, and the coulomb efficiency remained between 99% and 100%, demonstrating good cycle stability. The decrease in the cycle capacity was primarily due to the negative electrode, the volume of which changed greatly during the charging and discharging processes. Lattice distortion leads to a decrease in conductivity and shedding of active substances.

The unique performance of the NiO-rGO//AC system can be summarized as follows: (i) the presence of rGO can enhance conductivity, facilitate the transmission of ions and electrons, and reduce the lattice distortion of metal oxides caused by the insertion and extraction of lithium ions; (ii) a sufficient interface is provided by AC to accumulate opposing charges; and (iii) the proper ratio of positive to negative contributes to obtaining the best output performance.

### 3.4. The Prospect of Industrialization

Laboratory research into the development of new materials has been unable to keep pace with the needs of industry. Bottlenecks are caused by a variety of problems: the high cost of materials hampers large-scale production, the preparation and purification of certain materials produces toxic substances, there are difficulties with recycling used materials, etc. The research presented here highlights a number of opportunities to circumvent these barriers. Firstly, regarding cost, the prices of rGO and NiO are moderate. Secondly, regarding the preparation process, this study used the well-established Hummer method to prepare the rGO, while the NiO-rGO composite was prepared using the hydrothermal method and low-temperature heat treatment; these preparation processes have low environmental impacts and require little in the way of specialized equipment. This has the potential to lower production costs when scaled up to industrial production. Finally, the electrochemical performance of the system is excellent compared to established designs. Therefore, the LIC design presented in this paper offers many advantages when it comes to future commercial production.

## 4. Conclusions

In summary, an LIC was successfully designed using a NiO-rGO composite as the negative material and commercial AC as the positive material, producing a battery with excellent electrochemical performance. It was shown that the integration of NiO with carbon-based materials can improve the electrochemical performance in LIC applications. Furthermore, the presence of graphene enhances the transport of ions and electrons and effectively suppresses the volume expansion of the metal oxide when lithium ions are inserted into or extracted from the electrode material. In addition, the NiO-rGO composite featured a larger specific surface area and a nanoscale ion diffusion pathway, which was conducive to the insertion and extraction of lithium ions. The NiO-rGO composite had good reversible cycling performance at both low and high current densities, and also had a high reversible capacity at high current densities. In addition, the NiO-rGO//AC LIC system with a mass ratio of 1:2–1:4 (negative/positive) was synergistically fabricated. The LIC with a mass ratio of 1:3 achieved excellent electrochemical performance, with an energy density of 98.15 W h kg^−1^, a power density of 10.94 kW kg^−1^, a reasonable level of cycle stability, and a capacity retention of 72.1% after 10,000 cycles. The rationally designed NiO-rGO//AC system presents a number of superb opportunities in the development of high-performance LICs.

## Supplementary Materials

The following are available online at https://www.mdpi.com/article/10.3390/ma14133586/s1, Figure S1: Nyquist plots of NiO-rGO electrodes in the frequency range of 100 kHz–0.01 Hz and the inset is the enlarged EIS, Table S1: Electrochemical performances of recently published Li-ion hybrid capacitors in organic system.
