# Supplementary material for: Novel Lithium-Ion Capacitor Based on a NiO-rGO Composite
Source: Materials (Basel). 2021 Jun 27;14(13):3586. doi: 10.3390/ma14133586 (PMC8269625; doi:10.3390/ma14133586)
Supplement: Supplementary file 1 [file materials-14-03586-s001.zip › materials-1251433-supplementary.pdf]

materials

Article

Novel Lithium-Ion Capacitor Based on a NiO-rGO Composite

Qi An [1,*], Xingru Zhao [2], Shuangfu Suo [1] and Yuzhu Bai [1]

[1] Department of Mechanical Engineering, Tsinghua University, Beijing 100084, China; sfsuo@tsinghua.edu.cn (S.S.); baiyuzhu403@163.com (Y.B.)
[2] Beijing Institute of Nanoenergy and Nanosystems, Chinese Academy of Sciences, Beijing 101400, China; zhaoxingru@binn.cas.cn
* Correspondence: thaq@mail.tsinghua.edu.cn

demonstrated that the NiO-rGO composite, having been prepared following the aforementioned method, had a high crystal purity, with no other substances appearing.

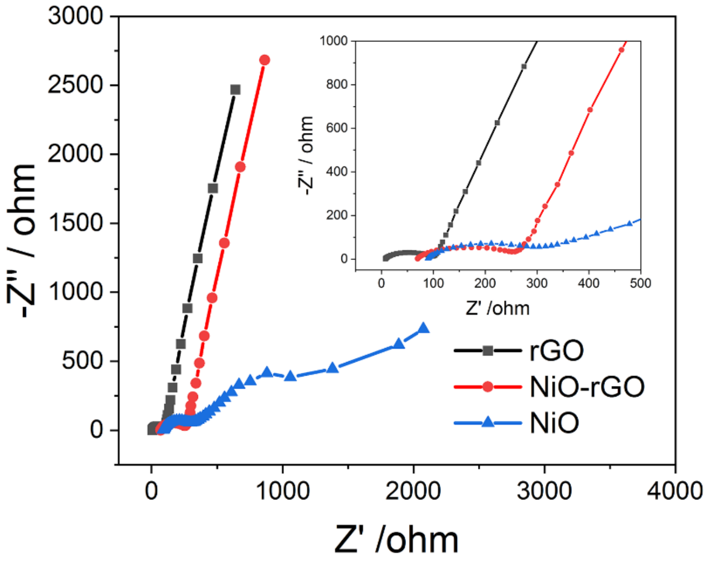

Figure S1. Nyquist plots of NiO-rGO electrodes in the frequency range of 100 kHz-0.01 Hz and the inset is the enlarged EIS**.**

Citation: An, Q.; Zhao, X.; Suo, S.; Bai, Y. Novel Lithium-Ion Capacitor Based on a NiO-rGO Composite. *Materials* **2021**, *14*, 3586. https://doi.org/10.3390/3586

Academic Editor: Suzy Surblé

Received: 23 May 2021
Accepted: 23 June 2021
Published: 27 June 2021

Publisher's Note: MDPI stays neutral with regard to jurisdictional claims in published maps and institutional affiliations.

Copyright: © 2021 by the authors. Submitted for possible open access publication under the terms and conditions of the Creative Commons Attribution (CC BY) license (http://creativecommons.org/licenses/by/4.0/).

Table S1. Electrochemical performances of recently published Li-ion hybrid capacitors in organic system.

Hybrid System (anode//cathode)	Energy density (Wh kg^{-1})	Power density kW kg^{-1}	Capacity retention //Cycing number	Working window (V)	Electrolyte	Ref.
TiO_2belt//Graphene	82	19	73%//600	0.0-3.8	$LiPF_6$-EC/DMC	1
NbN//NG	122.7	2	81.7%//1000	0.005-3.0	$LiPF_6$-EC/DMC	2
Activated carbon//carbonized polyimide microsphere	28.5	6.94 //	97.1%//5000	0.005-3.0	$LiPF_6$-EC/DMC	3
Carbon nanospheres//graphite	80	35.2	93%//4000	0.05-3.0	$LiPF_6$-EC/DMC	4
carbon //vanadiumnitride-rGO	162	10	83%//1000	0.01-3.0	$LiPF_6$-EC/DMC	5
carbon //MnO/CNS	184	15	76%//5000	0.0-2.7	$LiPF_6$-EC/DMC	6
AC//mesocarbon microbeads	92.3	5.5	97%//1000	0.005-3.0	$LiPF_6$-EC/DMC	7
TNb_2O_5/graphene//AC	47	18	93%//2000	0.8-3.0	$LiPF_6$	8
NiO/RGO//AC	32.5	19.78	92.7//3000	1.5	KOH	9
NiO-rGO//AC	98.15	10.94	72.1//10000	1.0-4.0	$LiPF_6$-EC/DMC/DEC	This work

Reference

1. Wang H, Guan C, Wang X, Fan HJ: A high energy and power Li-ion capacitor based on a TiO2 nanobelt array anode and a graphene hydrogel cathode. Small 2015, 11:1470-1477.
2. Liu M, Zhang L, Han P, Han X, Du H, Yue X, Zhang Z, Zhang H, Cui G: Controllable Formation of Niobium Nitride/Nitrogen-Doped Graphene Nanocomposites as Anode Materials for Lithium-Ion Capacitors. Part. Part. Syst. Charact. 2015, 32:1006-1011.
3. Han X, Han P, Yao J, Zhang S, Cao X, Xiong J, Zhang J, Cui G: Nitrogen-doped carbonized polyimide microsphere as a novel anode material for high performance lithium ion capacitors. Electrochim. Acta 2016, 196:603-610.
4. Yu X, Zhan C, Lv R, Bai Y, Lin Y, Huang Z-H, Shen W, Qiu X, Kang F: Ultrahigh-rate and high-density lithium-ion capacitors through hybriding nitrogen-enriched hierarchical porous carbon cathode with prelithiated microcrystalline graphite anode. Nano Energy 2015, 15:43-53.
5. Wang R, Lang J, Zhang P, Lin Z, Yan X: Fast and Large Lithium Storage in 3D Porous VN Nanowires-Graphene Composite as a Superior Anode Toward High-Performance Hybrid Supercapacitors. Adv. Funct. Mater. 2015, 25:2270-2278.
6. Wang H, Xu Z, Li Z, Cui K, Ding J, Kohandehghan A, Tan X, Zahiri B, Olsen BC, Holt CM, Mitlin D: Hybrid device employing three-dimensional arrays of MnO in carbon nanosheets bridges battery-supercapacitor divide. Nano Lett. 2014, 14:1987-1994.
7. Zhang J, Shi Z, Wang C: Effect of pre-lithiation degrees of mesocarbon microbeads anode on the electrochemical

performance of lithium-ion capacitors. Electrochim. Acta 2014, 125:22-28.
8. Kong L, Zhang C, Wang J, Qiao W, Ling L, Long D: Free-Standing T-Nb(2)O(5)/Graphene Composite Papers with Ultrahigh Gravimetric/Volumetric Capacitance for Li-Ion Intercalation Pseudocapacitor. ACS Nano 2015, 9:11200-11208.
9. Li Q, Wei Q, Xie L, Chen C, Lu C, Su F-Y, Zhou P: Layered NiO/reduced graphene oxide compositesby heterogeneous assembly with enhanced performance as high-performance asymmetric supercapacitor cathode. RSC Advances 2016, 6:46548-46557.
